# Association between the frequency of daily intellectual activities and cognitive domains: A cross‐sectional study in older adults with complaints of forgetfulness

**DOI:** 10.1002/brb3.1923

**Published:** 2020-11-03

**Authors:** Ai Iizuka, Hiroyuki Suzuki, Susumu Ogawa, Tomoya Takahashi, Sachiko Murayama, Momoko Kobayashi, Yoshinori Fujiwara

**Affiliations:** ^1^ Research Team for Social Participation and Community Health Tokyo Metropolitan Institute of Gerontology Tokyo Japan

**Keywords:** cognition, cognitive decline, forgetfulness, intellectual activity, MoCA

## Abstract

**Objectives:**

Frequent engagement in intellectual activities has been shown to reduce the risk of developing dementia. The present study sought to examine the association between the frequency of daily intellectual activities and cognitive domains in older adults with complaints of forgetfulness.

**Methods:**

A cross‐sectional study was conducted as a part of regional health examination in Tokyo from 2014 to 2016. A total of 436 participants were asked the frequency of intellectual activities in four categories: 1) reading, 2) writing, 3) using technology, and 4) watching TV and listening to the radio. The Japanese version of the Montreal Cognitive Assessment (MoCA‐J) scale was used for the cognitive assessments. The relationships between MoCA‐J scores and each intellectual activity were explored.

**Results:**

Binominal logistic regression analysis revealed that the frequencies of reading, writing, and using technology were significantly related to the language and attention, language, and memory domains, respectively, even after adjusting for demographic characteristics.

**Conclusions:**

The results suggested that the frequency of daily intellectual activities differed depending on the activity type, and each activity was related to a specific cognitive domain.

## Graphical abstract

Frequent engagement in intellectual activities has been shown to maintain or improve cognitive function. The present study sought to examine the association between the frequency of daily intellectual activities and cognitive domains in older adults with complaints of forgetfulness. The results suggested that the frequencies of reading, writing, and using technology were significantly related to cognitive domains of the language and attention, language, and memory, respectively.

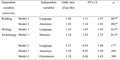

## 1 INTRODUCTION

Cognitive decline is a major problem in aging societies (Alzheimer's Disease International, 2013; Deary et al., 2009). Recently, a range of risk factors related to cognitive decline have been reported, including hypertension, diabetes, social isolation, and less frequent engagement in physical activities (Livingston et al., 2017). Early identification and modification of these risk factors may help prevent or delay the onset of dementia.

Because people with complaints of forgetfulness tend to have a higher risk of dementia, and poor cognitive function such as memory, attention, and language, (Benito‐León et al., 2010; Park et al., 2019; Weber et al., 2013) they are important targets for countermeasures against dementia. These individuals should be encouraged to actively participate in activities considered to be beneficial for preventing cognitive decline, including exercise and social activity. However, previous studies have reported that people with complaints of forgetfulness tend to be reluctant to participate in such activities (Wion et al., 2019; Teraoka et al., 2005). For people who are reluctant to participate in social activities, engaging in intellectual activities may be a viable option that is easy to implement and maintain.

Promoting intellectual activities among older adults is particularly important in aging societies, because less frequent engagement in intellectual activities is a major risk factor for cognitive decline (Livingston et al., 2017). Engagement in intellectual activities has been found to enhance synaptic transmission and neuroplasticity, (Richards & Deary, 2005) and to increase cognitive reserve (Stern & Munn, 2009; Stern, 2002, 2009). Several observational studies have reported that frequent engagement in intellectual activities in later life reduces the risk of developing dementia and maintains or improves cognitive function (Christensen & Mackinnon, 1993; Hultsch et al., 1993; Stern, 2009; Wilson et al., 2002; Wilson et al., 2007; Valenzuela & Sachdev, 2006; Yates et al., 2016). However, the relationship between the frequency of engagement in intellectual activities and cognitive function among people with complaints of cognitive decline have not been clarified. Elucidating this issue may help to identify better prevention strategies against cognitive decline.

Moreover, because previous studies have typically included various types of intellectual activity together, accurate assessment of the effect of each activity on cognitive function is difficult (Carlson et al., 2002; Iizuka et al., 2019; Verghese et al., 2006; Wang et al., 2006; Wang et al., 2012). For example, Wang et al. (2016) included playing board games, reading, writing, calligraphy or painting, playing music, drama or dancing, and listening to the radio together in the same category, “cognitive activities” (Wang et al., 2006). Similarly, Verghese et al. (2006) treated reading, writing, crossword puzzles, board or card games, group discussion, and playing music as “cognitive activities” (Wang et al., 2006). Because different types of intellectual activities have different characteristics, they may be associated with different cognitive domains (Verghese et al., 2006). Furthermore, for individuals with cognitive decline, the ease of implementation and continuation of activities is likely to differ between various types of activity. To clarify the effects of each activity on cognitive function, it is necessary to classify intellectual activities by careful consideration of the characteristics of each activity and to examine the specific relationships between each activity and each cognitive domain. When classifying intellectual activities, the most basic intellectual activities people engage in during daily life (daily intellectual activities) should be focused on first, because they constitute the basis of a vast range of intellectual activities.

The current study investigated the frequency of specific daily intellectual activities in older adults with complaints of forgetfulness and examined the association between the frequency of each activity and each cognitive domain.

## 2 METHODS

A cross‐sectional study was conducted as a part of a regional health examination organized by a local government in Toshima city, Tokyo, Japan from 2014 to 2016. The health examination comprehensively evaluated the health conditions of older adults with complaints of forgetfulness.

### 2.1 Ethical approval

Ethical approval was obtained from the Institutional Review Board and Ethics Committee of the Tokyo Metropolitan Institute of Gerontology (Acceptance No. 98, 2, 2014). We obtained written informed consent from all participants in this study.

### 2.2 Participants and data collection

Participants in the regional health examination were recruited by a public news magazine published by the local government. The purpose, methods, and ethical considerations of the study were explained to each participant in the health examination, after which, written informed consent was obtained. The inclusion criteria were as follows: aged 65 years or older, capable of independently performing activities of daily living (ADL), and having complaints of cognitive decline (e.g., a positive response to the question “Do you have complaints of forgetfulness?”). Individuals with a history of stroke or mental illness were excluded.

### 2.3 Measures

We obtained demographic information from participants, including age, sex, duration of education, and past medical history, using self‐report questionnaires at the site of the health examinations. Three categories were created for the variable of education depending on the years of formal education received: junior high school or less (9 years or less), high school (10–12 years), and university (13 years or more). The Japanese version of the Montreal Cognitive Assessment (MoCA‐J) scale ( Fujiwara et al., 2010) was used for assessing cognitive domains, including memory, language, executive function, attention, visuospatial abilities, and orientation.

#### 2.3.1 The frequency of daily intellectual activities

We regarded daily intellectual activities as common activities in which seeking or processing information played a central role and which had minimal physical demands or social requirements (Wilson et al., 2007).

First, we developed a questionnaire and asked about the frequency of the following daily intellectual activities: reading newspaper, reading magazine, reading books, entries in a diary, writing letters, using computers, using cell phones, watching TV, and listening to the radio. The participants selected the most accurate response on a six‐point Likert‐type scale (0 = Never, 1 = Once a year or less, 2 = Several times a year, 3 = Several times a month, 4 = Several times a week, or 5 = almost every day). Since the focus of this study was daily intellectual activities, housework and special activities, such as leisure activities, were excluded.

Next, we carefully examined the characteristics of the activities and divided them into passive activities (watching TV and listening to the radio) and active activities (others). We also divided active activities into three categories based on modality: (a) reading letters with eyes (Reading): reading newspapers, books, and magazines; (b) writing with hands (Writing): writing letters and entries in a diary; and (c) operating machines (Technology): using computers and cell phones. Then, the maximum frequency was calculated in each category.

#### 2.3.2 Cognitive function

Besides memory, we also focused on other cognitive domains including language, attention, visuospatial ability, and orientation, as there is a possibility that complaints of forgetfulness are also affected by declining of other cognitive domains. Therefore, we used the MoCA‐J, which is capable of evaluating cognitive domains in addition to memory and global cognition in this study. The score range was 0–30, with a cutoff score of 25/26 for mild cognitive impairment. The MoCA‐J assesses six cognitive domains: (a) memory; (b) language; (c) executive functions; (d) attention; (e) visuospatial abilities; and (f) orientation.

The details of the examination and correspondence to each cognitive domain are described below.

##### Memory

Memory was assessed using an immediate and delayed recall task. Participants memorized five items and attempted to recall them after approximately 5 min. The score range was 0–5.

##### Language

Language was assessed using three items: (a) a naming task with low‐familiarity animals (lion, rhinoceros, camel) (3 points), (b) repetition of two complex sentences (2 points), and (c) a phonic verbal fluency task using a specific Japanese syllable, “Ka” (1 point). The score range was 0–6.

##### Executive functions

Executive functions were assessed using three items: (a) an alternating trail‐making (1 point), (b) a phonemic verbal fluency task (the same task used in the “language” category) (1 point), and (c) a two‐item verbal abstraction task to explain what each pair of words has in common (2 points). The score range was 0–4.

##### Attention

Attention was assessed using three items: (a) a sustained attention task (1 point), (b) a serial subtraction task (3 points), and iii) a digit span task (digits forward [1 point] and backward [1 point]). The score range was 0–6.

##### Visuospatial abilities

Visuospatial abilities were assessed using two items: (a) a clock‐drawing task (3 points), and (b) a three‐dimensional cube copy task (1 point). The score range was 0–4.

##### Orientation

Orientation to time and place was assessed by asking about the time and location of the examination. The score range was 0–6.

### 2.4 Statistical analysis

#### 2.4.1 Distribution of the frequency of daily intellectual activities

To present the distribution of the frequency of engagement in the intellectual activity by classified category, we calculated the percentage of participants who answered in each of the six frequency options.

Based on the results, participants were divided into “high‐frequency” and “low‐frequency” groups for each activity.

#### 2.4.2 Association between the frequency of daily intellectual activities and cognitive domains

To investigate participants' characteristics by frequency group, we compared basic characteristics and MoCA‐J scores between groups. Two independent samples, *t* tests, were used to compare age and MoCA‐J total scores, chi‐square tests were used to compare sex and education, and Mann–Whitney *U* tests were used to compare the scores of each cognitive domain.

Associations between the frequency of engagement in each activity and cognitive domains which were observed significant differences in the Mann–Whitney *U* tests were analyzed using binomial logistic regression analysis. We set the frequency of activity (high = 1, low = 0) as the dependent variable and each cognitive domain as the independent variables in separate logistic models. Cognitive domains were adjusted for age, sex, and education.

A *p*‐value <.05 was considered statistically significant. All analyses were conducted using SPSS (version 23; IBM Inc.).

## 3 RESULTS

### 3.1 Participants' characteristics

A total of 436 participants (73.9% female) who met the inclusion criteria were included in the analysis. Participants' basic characteristics are shown in Table 1. Participants' mean age ± standard deviation was 74.1 ± 5.8 years, and their mean duration of education was 13.3 ± 2.4 years.

**Table 1:** Participants' basic characteristics and MoCA‐J scores. (*n* = 436)

		Mean	*SD*
Age	Years	74.1	5.8
Education	Years	13.3	2.4
MoCA‐J (total score)	/30	24.5	3.2
Memory	/5	2.8	1.7
Language	/6	4.4	1.0
Executive function	/4	3.0	0.9
Attention	/6	4.8	1.2
Visuospatial abilities	/4	3.6	0.6
Orientation	/6	5.6	0.7

Abbreviations: MoCA‐J, Japanese version of the Montreal Cognitive Assessment; *SD*, standard deviation.

### 3.2 Distribution of the frequency of daily intellectual activities

The distribution frequency for each category of daily intellectual activity is shown in Table 2 (Note that, the frequency of activity before classification is shown in Appendix A).

**Table 2:** Distribution of the frequency of daily intellectual activities. (*n* = 436)

Activity	Frequency	*N*	%
Reading	Almost every day	374	85.8
	Several times a week	41	9.4
	Several times a month	16	3.7
	Several times a year	2	0.5
	Once a year or less	1	0.2
	Never	2	0.5
Writing	Almost every day	138	31.7
	Several times a week	62	14.2
	Several times a month	57	13.1
	Several times a year	88	20.2
	Once a year or less	29	6.7
	Never	62	14.2
Technology	Almost every day	221	50.7
	Several times a week	87	20.0
	Several times a month	35	8.0
	Several times a year	9	2.1
	Once a year or less	2	0.5
	Never	82	18.8
Engaging in passive activity	Almost every day	430	98.6
	Several times a week	4	0.9
	Several times a month	1	0.2
	Several times a year	1	0.2
	Once a year or less	0	0.0
	Never		

Abbreviations: N, number of participants.

In total, 98.6% of participants reported watching TV and listening to the radio almost every day, while more than half reported reading or using technology almost every day. Regarding writing, the frequency was widely distributed: almost every day, 31.7%; several times a week, 14.2%; several times a month, 13.1%; several times a year, 20.2%; less than once a year, 6.7%; and never, 14.2%.

For each activity, the participants were divided into “high‐frequency” and “low‐frequency” frequency groups based on the median values.

The mean cognitive domain scores and the results of the analysis comparing the high‐ and low‐frequency groups for each activity are shown in Tables 3, 4, 5. Regarding reading, language and attention were significantly higher in the high‐frequency group compared with the low‐frequency group (both *p* < .01). Regarding writing, language was significantly higher in the high‐frequency group compared with the low‐frequency group (*p* < .01). Regarding technology, MoCA‐J total scores, memory, language, attention, and orientation were higher in the high compared with the low‐frequency group (all *p* < .05). Regarding watching TV and listening to the radio, because 98.6% of participants reported engaging in these activities almost every day, we did not consider it necessary to perform a statistical analysis.

**Table 3:** Participants' characteristics and MoCA‐J scores by frequency group (reading)

Activity	*N* (high/low)	Variables	High	Low	*p*
			Mean (*SD*)		
Reading	374/62	Age	74.94 (5.89)	74.06 (5.79)	.270
		Sex (*N*: male/female)	266/108	6/56	.001*
		Education (*N*: a/b/c)	10/36/26	26/141/207	<.001*
		MoCA‐J (total score)	24.56 (3.16)	23.77 (3.32)	.073
		Memory	2.76 (1.65)	2.69 (1.67)	.806
		Language	4.51 (0.95)	4.06 (1.09)	.004*
		Executive function	2.98 (0.91)	2.79 (1.05)	.234
		Attention	4.83 (1.12)	4.23 (1.29)	<.001*
		Visuospatial abilities	3.58 (0.64)	3.48 (0.64)	.171
		Orientation	5.61 (0.64)	5.55 (0.73)	.607

Note

a: junior high school or less (9 years or less).

b: high school (10–12 years).

c: university (13 years or more).

Abbreviations: High, high‐frequency group; low, low‐frequency group; MoCA‐J, Japanese version of the Montreal Cognitive Assessment; *N*, number of participants; *SD*, standard deviation.

* *p* < .05.

**Table 4:** Participants' characteristics and MoCA‐J scores by frequency group (writing)

Activity	*N* (high/low)	Variables	High	Low	*p*
			Mean (*SD*)		
Writing	200/236	Age	74.39 (5.87)	74.01 (5.76)	.501
		Sex (*N*: male/female)	42/158	72/164	.029*
		Education (*N*: a/b/c)	21/100/115	15/77/108	.537
		MoCA‐J (total score)	24.68 (3.17)	24.25 (3.21)	.166
		Memory	2.82 (1.70)	2.69 (1.62)	.362
		Language	4.58 (0.96)	4.33 (0.99)	.006*
		Executive function	3.05 (0.89)	2.87 (0.97)	.056
		Attention	4.79 (1.13)	4.72 (1.20)	.657
		Visuospatial abilities	3.53 (0.64)	3.59 (0.63)	.311
		Orientation	5.64 (0.59)	5.57 (0.70)	.438

Note

a: junior high school or less (9 years or less).

b: high school (10–12 years).

c: university (13 years or more).

Abbreviations: High, high‐frequency group; low, low‐frequency group; MoCA‐J, Japanese version of the Montreal Cognitive Assessment; *N*, number of participants; *SD*, standard deviation.

* *p* < .05.

**Table 5:** Participants' characteristics and MoCA‐J scores by frequency group (technology)

Activity	*N* (high/low)	Variables	High	Low	*p*
			Mean (*SD*)		
Technology	215/221	Age	72.79 (5.36)	75.61 (5.92)	<.001*
		Sex (*N*: male/female)	60/161	54/161	.664
		Education (*N*: a/b/c)	22/92/101	14/85/122	.139
		MoCA‐J (total score)	24.98 (3.04)	23.90 (3.26)	<.001*
		Memory	3.05 (1.50)	2.44 (1.74)	<.001*
		Language	4.55 (0.94)	4.33 (1.01)	.043*
		Executive function	3.01 (0.94)	2.89 (0.92)	.113
		Attention	4.87 (1.11)	4.62 (1.22)	.045*
		Visuospatial abilities	3.52 (0.67)	3.60 (0.61)	.200
		Orientation	5.67 (0.59)	5.53 (0.70)	.022*

Note

a: junior high school or less (9 years or less).

b: high school (10–12 years).

c: university (13 years or more).

Abbreviations: High, high‐frequency group; low: low‐frequency group; MoCA‐J, Japanese version of the Montreal Cognitive Assessment; *N*, number of participants; *SD*, standard deviation.

* *p* < .05.

### 3.3 Association between the frequency of daily intellectual activities and cognitive domains

The results of the binomial logistic regression analysis in which each cognitive domain was set as the independent variable are shown in Table 6. The analysis revealed that the frequency of reading was significantly related to language (odds ratio [OR]: 1.48, 95% confidence interval [CI]: 1.11–1.97; *p* = .007) and attention (OR: 1.45, 95% CI: 1.14–1.83; *p* = .002), even after adjusting for age, sex, and educational history. In addition, the frequency of writing was found to be significantly related to language (OR: 1.29, 95% CI: 1.05–1.59; *p* = .012) and that of technology was found to be significantly related to memory (OR: 1.18, 95% CI: 1.03–1.35; *p* = .013).

**Table 6:** Results of binominal logistic regression analysis^a^

Dependent variables (activity)	Independent variables	Odds ratio (exp [B])	95% CI		*p*
Reading					
Model 1	Language	1.48	1.11	1.97	.007*
Model 2	Attention	1.45	1.14	1.83	.002*
Writing					
Model 1	Language	1.29	1.05	1.59	.012*
Technology					
Model 1	Memory	1.18	1.03	1.35	.013*
Model 2	Language	1.15	0.93	1.40	.177
Model 3	Attention	1.10	0.92	1.30	.268
Model 4	Orientation	1.18	0.86	1.63	.300

^a^ Adjusted by age, sex and educational category.

* *p* < .05.

## 4 DISCUSSION

The current study aimed to clarify the frequency of daily intellectual activities by type in older adults with complaints of forgetfulness and to examine the associations between the frequency of each activity and specific cognitive domains. The results suggested that the frequency of daily intellectual activities differed depending on the type of activity. The frequencies of reading, writing, and using technology were significantly related to the language and attention, language, and memory domains, respectively.

More than 80% of participants reported that they read books, magazines, or newspapers frequently, and an association was observed between reading frequency and language and attention. Reading sentences requires the reader to interpret many words and different grammatical forms, supporting the finding of a relationship between reading and language ability. Furthermore, reading long sentences requires the reader to attend to written letters for a relatively long duration. Therefore, it is also conceivable that sustained attention is related to reading.

The results revealed an association between the frequency of writing and language. Writing letters and entries in a diary requires the generation of words and sentences; this was considered to correspond to the “verbal fluency” word recall task in the MoCA‐J, which examines language function. We speculate that writing in a diary may be related to memory function, because doing so requires recall of past episodes. However, no significant effects related to memory were found in this study.

Regarding the use of technology, significant association was observed in memory. Previous studies have reported that learning new technologies can improve memory function, (Klusmann et al., 2010; Park et al., 2014) and complex engagement with technology can contribute to the maintenance and even enhancement of memory (Hultsch et al., 1999). The results of the present study support these previous findings.

According to the frequency distribution of each activity, 98.6% of participants reported watching TV and listening to the radio every day. Based on this result, we did not include the category in the logistic regression analysis, for two reasons. First, it was inappropriate to conduct statistical analysis. Second, from the questionnaire it was unclear whether participants actively watched TV or/listened to radio programs, or if they simply left the TV or radio turned on. To include TV and radio in the analysis, this difference needs to be clarified in future studies.

The present study involved several limitations that should be considered. First, because the study was conducted with people with complaints of forgetfulness, it may not necessarily reflect the characteristics of the general population. Second, the results may differ in developing countries because the participants in the present study had a relatively high duration of education. Third, in the present study, we examined the relationships between various types of daily intellectual activities and specific cognitive domains; these results may become clearer by detailed analysis that includes the amount of exposure time to activities.

Finally, as this was a cross‐sectional study, two potential interpretations of the results should be considered. First, engaging in daily intellectual activities at a high frequency may be difficult unless the specific cognitive domain corresponding to each activity is maintained. Second, it is possible that a specific cognitive domain may be maintained and improved by corresponding intellectual activities. From this perspective, a person with impairment in a specific cognitive domain may be able to improve function by frequently engaging in activities that correspond to that domain. To further elucidate the association between daily intellectual activities and cognitive domains, longitudinal and intervention studies should be conducted in the future.

## 5 CONCLUSION

The results of the present study suggested that the frequency of daily intellectual activities in older adults with complaints of forgetfulness differed depending on activity type. In addition, the frequencies of reading, writing, and using technology were significantly related to the language and attention, language, and memory domains, respectively. These results demonstrate that daily intellectual activities are related to specific cognitive domains.

## CONFLICT OF INTEREST

The authors have no potential conflicts of interest to declare with respect to the research, authorship, and/or publication of this article.

## AUTHOR CONTRIBUTIONS

Ai Iizuka was responsible for developing the study concept and design, analyzed the data, and drafted the manuscript. Hiroyuki Suzuki contributed making the study concept and design, analysis, and interpretation of data. Susumu Ogawa assisted acquisition, analysis, and interpretation of data. Tomoya Takahashi and Sachiko Murayama contributed acquisition of the participants and data. Momoko Kobayashi assisted drafting manuscript. Yoshinori Fujiwara contributed to the critical revision of the manuscript, supervised the development of the research question, and assisted research accomplishment. All authors approved the final manuscript.

### Peer Review

The peer review history for this article is available at https://publons.com/publon/10.1002/brb3.1923.

## Data Availability

The data that support the findings of this study are available from the corresponding author upon reasonable request.

## Appendix A Distribution of frequency of participation in daily intellectual activity (n = 436)

**Table:**

Activity	Frequency (*N* (%))					
	About everyday	Several times a week	Several times a month	Several times a year	Once a year or less	Never
Reading	374 (85.8)	41 (9.4)	16 (3.7)	2 (0.5)	1 (0.2)	2 (0.5)
Reading news paper	354 (81.2)	42 (9.6)	19 (4.4)	4 (0.9)	1 (0.2)	16 (3.7)
Reading magazine	78 (17.9)	151 (34.6)	132 (30.3)	54 (12.4)	6 (1.4)	15 (3.4)
Reading books	113 (25.9)	98 (22.5)	109 (25.0)	60 (13.8)	24 (5.5)	32 (7.3)
Writing	138 (31.7)	62 (14.2)	57 (13.1)	88 (20.2)	29 (6.7)	62 (14.2)
Entries in a diary	134 (30.7)	60 (13.8)	24 (5.5)	15 (3.4)	4 (0.9)	199 (45.6)
Writing letters	9 (2.1)	18 (4.1)	86 (19.7)	185 (42.4)	52 (11.9)	86 (19.7)
Technology	221 (50.7)	87 (20.0)	35 (8.0)	9 (2.1)	2 (0.5)	82 (18.8)
Using computers	113 (25.9)	59 (13.5)	36 (8.3)	14 (3.2)	2 (0.5)	212 (48.6)
Using cell phones	170 (39.0)	88 (20.2)	53 (12.2)	11 (2.5)	5 (1.1)	109 (25.0)
Engaging in passive activity	430 (98.6)	4 (0.9)	1 (0.2)	0 (0.0)	1 (0.2)	0 (0.0)
Watching TV	422 (96.8)	9 (2.1)	2 (0.5)	0 (0.0)	1 (0.2)	2 (0.5)
Listening to the Radio	176 (40.4)	39 (8.9)	47 (10.8)	39 (8.9)	7 (1.6)	128 (29.4)

Abbreviation: N, number of participants.
